# The Role of Peroxisome Proliferator-Activated Receptors in Kidney Diseases

**DOI:** 10.3389/fphar.2022.832732

**Published:** 2022-03-04

**Authors:** Jianjun Gao, Zhaoyan Gu

**Affiliations:** ^1^ Department of Nephrology, Chinese PLA Strategic Support Force Characteristic Medical Center, Beijing, China; ^2^ Department of Endocrinology, Second Medical Center, Chinese PLA General Hospital, Beijing, China; ^3^ National Clinical Research Center for Geriatric Diseases, Chinese PLA General Hospital, Beijing, China

**Keywords:** peroxisome proliferator-activated receptors, kidney disease, acute kidney injury, diabetic nephropathy, chronic kidney disease

## Abstract

Peroxisome proliferator-activated receptors (PPARs) are members of the nuclear hormone receptor superfamily of ligand-activated transcription factors. Accumulating evidence suggests that PPARs may play an important role in the pathogenesis of kidney disease. All three members of the PPAR subfamily, PPARα, PPARβ/δ, and PPARγ, have been implicated in many renal pathophysiological conditions, including acute kidney injury, diabetic nephropathy, and chronic kidney disease, among others. Emerging data suggest that PPARs may be potential therapeutic targets for renal disease. This article reviews the physiological roles of PPARs in the kidney and discusses the therapeutic utility of PPAR agonists in the treatment of kidney disease.

## Introduction

Peroxisome proliferator-activated receptors (PPARs), a group of nuclear hormone receptors, consist of threeisotypes, i.e., PPARα, PPARβ/δ, and PPARγ (Xi et al., 2020). PPARs can regulate gene transcription in either ligand-dependent or -independent manner. The target genes are critical for fatty acid oxidation (FAO) and transportation, glucose metabolism, adipogenesis, cholesterol transportation and biosynthesis, apoptosis, and the inflammatory response (Tyagi et al., 2011; Derosa et al., 2018). Numerous studies employing experimental and clinical models have shown that PPARs play important roles in lipid metabolism and energy homeostasis in the kidney (Tovar-Palacio et al., 2012; Corrales et al., 2018) (Table 1). This review focuses on the roles of PPARs in renal metabolism as well as therapeutic strategies targeting the activation of PPARs in kidney disease.

**TABLE 1:** PPAR subtypes, chromosome location, expression site, and their functions in kidney diseases.

Subtype	Chromosome	Site of expression in renal	Functions
PPARα	3	Proximal tubules	Increase fatty acid oxidation
			Anti-inflammation
		Medullary thick ascending limbs	Anti-apoptosis
			Anti-necrosis
		Podocytes	Attenuate albuminuria
			Improve insulin resistance
		Glomerular mesangial cells	Against oxidative stress
			Against glomerular and tubulointerstitial fibrosis
PPARβ	22	Glomerular mesangial cells	Increase fatty acid oxidation
		Proximal tubule of the cortex and medulla	Anti-inflammation
		Stromal cells	Against oxidative stress
			Immune compatibility
PPARγ	6	Distal medullary collecting ducts	Fatty acid oxidation
			Increase glucose metabolism
		Proximal and distal tubules	Decrease insulin resistance
			Anti-inflammation
		Glomerular mesangial cells	Anti-apoptosis
			Antioxidant
		Podocytes	Activate autophagy
			Against tubulointerstitial fibrosis
		Renal vasculature	Regulate immune system

## PPAR Family

**FIGURE 1:**
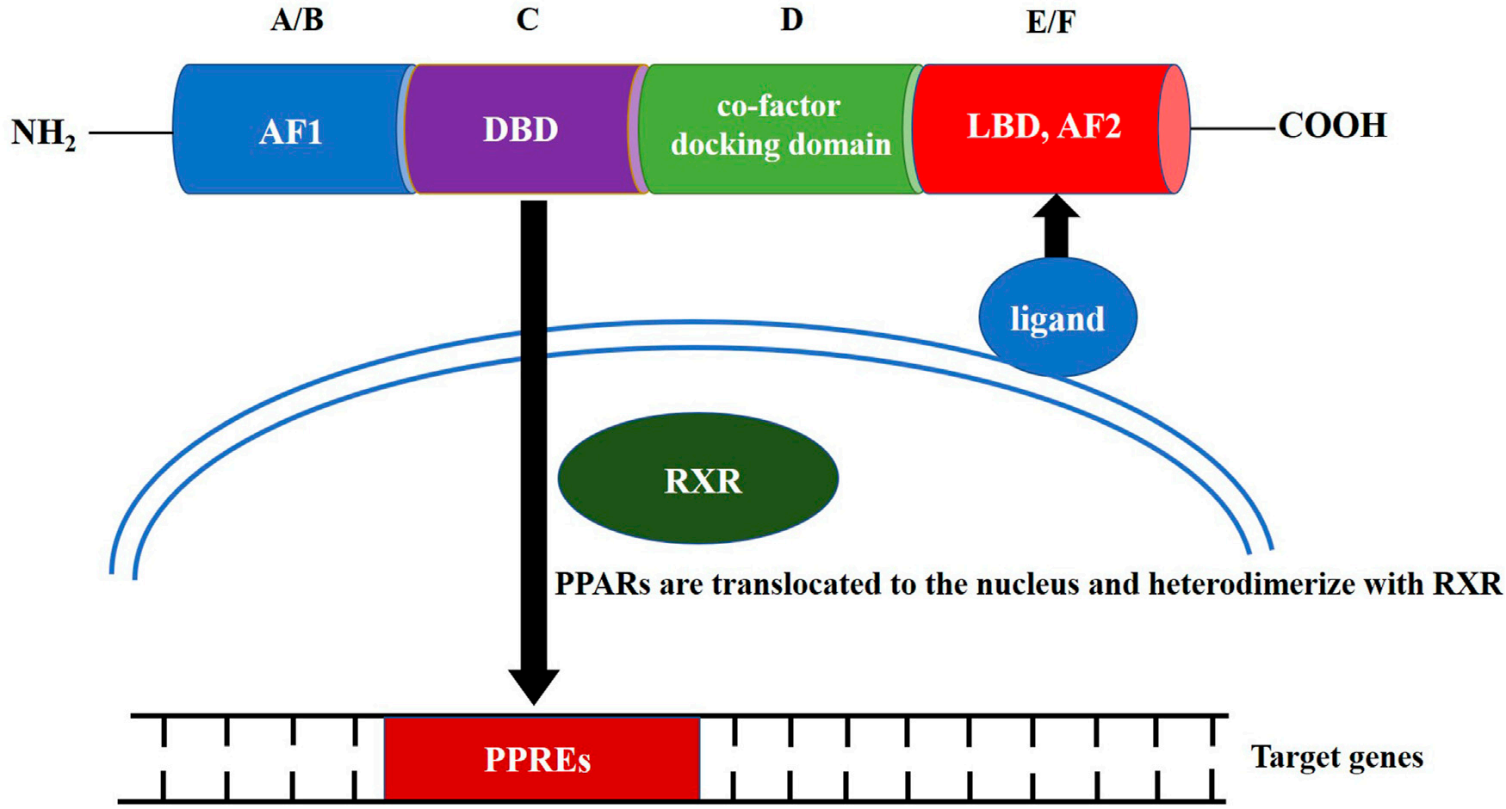
Structure and molecular mechanism of action of peroxisome proliferator-activated receptors.

PPAR family proteins have four main functional segments: the N-terminal ligand-independent transactivation domain (AF1, A/B domain), DNA-binding domain (DBD or C domain), co-factor docking domain (D domain), and C-terminal E/F domain that includes the ligand-binding domain (LBD) and ligand-dependent transactivation domain (AF2 domain; Figure 1) (Miyachi, 2021). The LBD and C-terminal activation functional domain form a large ligand-binding pocket. After the ligand-binding pocket interacts with a ligand, PPARs are translocated to the nucleus, either homodimerize or heterodimerize with another nuclear receptor, the retinoid X receptor (RXR) (Grygiel-Górniak, 2014). The PPRA or PPAR/RXR dimer binds to specific DNA response elements, peroxisome proliferator response elements (PPREs), to activate gene transcription. The conserved DBD is the domain involved in binding to PPREs (Berger and Moller, 2002). PPREs most commonly consist of a direct repeat of hexameric core recognition elements with a 1-bp spacer (DR1, 5′-AGGTCANAGGTCA-3′) located in the promoter regions of PPAR target genes (Dixon et al., 2021). After activation of the PPAR/RXR complex at the PPRE, the PPAR/RXR heterodimer can recruit diverse nuclear receptor co-factors, including coactivators, such as PPARγ coactivator-1α (PGC-1α), or co-repressors, such as the nuclear co-repressor and the silencing mediator for retinoid and thyroid hormone receptors (Dowell et al., 1999; Qi et al., 2000; Petr et al., 2018).

Although PPARs show a high degree of identity at the amino acid level and very similar structures, each isoform has unique tissue distribution, ligand selectivity, and biological functions (Ruan et al., 2008). The selectivity of each of the three PPAR isotypesis dependent on the divergent amino-acid sequences in the LBD. Fatty acids and their metabolites, synthetic pharmaceutical agents, including hypolipidemic fibrates, and the antidiabetic agent thiazolidinedione (TZD) have been shown to bind to and activate PPAR (Ricote et al., 2004; Monsalve et al., 2013). In recent years, many PPAR-null mice models have been available to study their implicaton in kidney physiology (Table 2).

**TABLE 2:** Physiological and pathological implications in kidney of PPAR-null mice for the different isotypes.

Isotypes of PPAR-null mice	Physiological and pathological implication
PPARα-null	• PPARα-null mice exhibited significantly greater kidney dysfunction after I/R injury, as assessed by higher serum creatinine levels and enhanced tubular necrosis Portilla et al. (2000)
	• PPARα-null mice had worse kidney function and metabolic derangement in experimental polymicrobial sepsis. Tissue mRNA expression of markers of kidney injury and inflammation were more elevated. Expression of enzymes associated with FAO and fatty acid transport was lower Iwaki et al. (2019)
	• Diabetic PPARα-null mice exhibited increased blood glucose, HbA1c, serum free fatty acid and triglyceride levels, and a persistent increase in urine albumin excretion. The increase in type IV collagen and TGF-β in the glomeruli were more prominent in diabetic PPARα-null mice Panchapakesan et al. (2004)
	• Aged PPARα null mice showed reduced expression of FAO-associated proteins and genes, higher lipid accumulation, vacuoles in tubules compared to control littermates Chung et al. (2018)
PPARβ-null	• PPARβ-null mice developed more severe ischemic acute renal failure than wild-type mice. Epithelial cell sloughing was more extensive in PPARβ-null mice, leading to tubular dilation and cast formation Lee et al, (2011)
	• PPARβ/δ-null female mice showed impairment of apoptotic cell clearance and reduction in anti-inflammatory cytokine production. These mice were much more likely to develop autoimmune kidney disease, a lupus-like autoimmunity Mukundan et al. (2009)
PPARγ-null	• PPARγ null mice showed increased glucosuria and albuminuria. With age, the mice developed renal insufficiency, advance of type 2 diabetes, and APS Toffoli et al. (2017)
	• The proximal tubular epithelial cells PPAR-γ deletions mice developed more severe tubulointerstitial fibrosis Zhao et al, (2016)

## PPARα

PPARα, the first member of the PPAR subfamily identified, is highly expressed in tissues that exhibit high levels of mitochondrial and FAO activity, including those of the liver, kidney, intestinal mucosa, and heart (Dixon et al., 2021). Lower levels of PPARα expression have also been detected in several other tissues. Within the kidney, PPARα is abundant in the proximal tubules and medullary thick ascending limbs, with much lower levels in glomerular mesangial cells (Guan et al., 1997; Kamijo et al., 2002). Given the high levels of expression in proximal tubules and medullary thick ascending limbs, PPARα has been implicated in metabolic regulation of the kidney. Many types of fatty acids and synthetic lipid-lowering fibrates (e.g., fenofibrate, clofibrate) can serve as PPARα agonists and regulate the transcription of several genes involved in FAO and the inflammatory response in the kidney (Di Paola and Cuzzocrea, 2007). It has been reported that activation of PPARα by clofibrate significantly induced the expression of β-oxidation enzymes in the renal cortex, including long-chain acyl-CoA dehydrogenase, medium-chain acyl-CoA dehydrogenase, and acyl-CoA oxidase (Ouali et al., 1998). PGC-1α, a coactivator of PPARα, is one of the main upstream transcriptional regulators of mitochondrial biogenesis and activity (Wu et al., 1999; Arany et al., 2005). In the kidney, PGC-1α is predominantly expressed in proximal tubules and medullary thick ascending limbs. Recent evidence supports the suggestion that PPARα and PGC-1α are critical regulators of the kidney involved in maintaining the balance of energy production and consumption (Portilla, 2003; Chambers and Wingert, 2020).

### PPARα and Acute Kidney Injury

PPARα-mediated FAO has been suggested to play an important regulatory role in the pathogenesis of acute kidney injury (AKI). PPARα null mice subjected to ischemia/reperfusion (I/R) injury exhibited significantly enhanced cortical necrosis and poorer kidney function in comparison to wild-type controls (Portilla et al., 2000). Upon cisplatin-induced AKI, the binding of PPARα to its target genes was inhibited and the expression of its coactivator, PGC-1α, was decreased in the mouse kidney and proximal tubule cells in culture, suggesting that FAO is suppressed in cisplatin-induced AKI (Portilla et al., 2002). Mice deficient in PPARα have poorer kidney function with sepsis-induced AKI, which is also related to reduced FAO and increased inflammation (Iwaki et al., 2019). Clinical data indicated that genome-wide expression profiles are characterized by repression of the PPARα signaling pathway with increased incidence of severe sepsis in AKI (Wong et al., 2009). Lipopolysaccharide (LPS)-treated mice exhibited a 40% decrease in renal FAO and inhibition of the expression of key transcription factors required for FAO. LPS also caused reductions in renal PPARα, RXR, and PGC-1α mRNA levels (Feingold et al., 2008). PPARα exhibits a protective role against sepsis-associated AKI by improving reduced FAO and increased inflammation (Iwaki et al., 2019). The increased expression of PPARα in proximal tubular epithelial cells in mice was shown to be sufficient to maintain FAO and protect kidney function and morphology in AKI (Li et al., 2009). PPARα ligands attenuate cisplatin-induced AKI by preventing the inhibition of FAO (Li et al., 2004a), reducing apoptosis and necrosis in proximal tubule cells through a decrease in endonuclease G activity (Li et al., 2004b), and limiting inflammatory processes by blocking NF-κB activity (Li et al., 2005; Baud and Letavernier, 2007). The activation of NF-κB, apoptosis, and oxidative stress induced by fatty acid-bound albumin (FA-BSA) in HK-2 cells was also markedly suppressed by fenofibrate (Zuo et al., 2015). Therefore, PPARα may be considered as a novel therapeutic target for preventing AKI. The mitochondrial matrix protein cyclophilin D (CypD) binds to PPARα and inhibits its nuclear translocation as well as the transcription of PPARα-regulated FAO genes during cisplatin-induced AKI. The genetic or pharmacological inhibition of CypD was reported to preserve PPARα transcriptional activity and prevent FAO impairment (Jang et al., 2020). The upregulation of PGC-1α can improve FAO and renal recovery from I/R injury by regulating NAD biosynthesis (Tran et al., 2016). Therefore, the regulation of PGC-1α was also identified as a new way to improve AKI.

### PPARα and Diabetic Nephropathy

Recent studies suggested important roles of FAO dysfunction and insulin resistance in the pathogenesis and progression of diabetic nephropathy (DN). PPARα may be a promising therapeutic target for treating diabetic renal complications. Herman et al. reported heavy lipid deposition and increased amounts of intracellular lipid droplets in kidney biopsies of patients with DN. It is also worth noting that several genes involved in FAO pathways, including PPARα, were downregulated (Herman-Edelstein et al., 2014). In an experimental study, more severe glomerular structural changes as well as albuminuria were noted in diabetic PPARα-knockout mice, with increased type IV collagen and transforming growth factor (TGF)-β expression detected in the glomerular lesions (Park et al., 2006). The PPARα agonist fenofibrate has been shown to reduce fasting blood glucose and insulin resistance, decrease urinary albumin excretion and glomerular mesangial expansion, suppress oxidative stress, and attenuate inflammation in diabetic animals (Park et al., 2006; Zuo et al., 2015; Yaribeygi et al., 2018). In addition, fenofibrate can downregulate the TGF-β signaling pathway, which plays a key role in the progression of DN (Wilmer et al., 2002). Endothelial dysfunction-induced M1 macrophage recruitment has been shown to play a key role in the development of DN. Fenofibrate can prevent DN by reducing M1 macrophage recruitment through regulating endothelial cell function as observed in a mouse model of type 2 diabetes (Feng et al., 2021). The PPARα activator gemfibrozil not only alleviated dyslipidemia but also attenuated albuminuria in normotensive noninsulin-dependent diabetic patients (Smulders et al., 1997). The Action to Control Cardiovascular Risk in Diabetes (ACCORD) study additionally demonstrated that fibrate therapy with intensive glucose control could significantly reduce microalbuminuria and macroalbuminuria in patients with type 2 diabetes (Ginsberg et al., 2010; Ismail-Beigi et al., 2010). It was reported that the downregulation of PGC-1α significantly increased reactive oxygen species in high glucose-stimulated renal mesangial cells, and endogenous PGC-1α expression resulted in protective effects against oxidative stress, glomerulosclerosis, and tubulointerstitial fibrosis in experimental DN (Zhang et al., 2018).

### PPARα and Chronic Kidney Disease

PPARα was shown to be downregulated in aggressive mouse models of autosomal dominant polycystic kidney disease (ADPKD) and primary human ADPKD cells, suggesting that decreased PPARα function may underlie the impaired FAO and oxidative phosphorylation in ADPKD (Hajarnis et al., 2017; Lakhia et al., 2018).

Meanwhile, PPARα also regulates age-associated renal fibrosis. PPARα and FAO-associated gene expression was decreased with age, which is directly related to the lipid metabolic disturbances in renal diseases in aged (Tovar-Palacio et al., 2012). PPRAα null mice exhibited higher lipid accumulation in renal tubule compared with littermates, which suggested the importance of PPRAα in the development of age-related renal fibrosis (Chung et al., 2018). Targeting PPARα is also useful for preventing age-associated CKD.

In unilateral ureteral obstruction models, preserving the expression of PPARα led to a reduction in tubulointerstitial fibrosis and inflammation. Further analyses reveal decreased production of TGF-β, IL-1b, IL-6, and TNF-α, reduced macrophage infiltration (Li et al., 2013). PPARα also plays an important role in glomerulonephritis. Saga et al. reported that bezafibrate, a PPARα agonist, attenuated the severity and extent of diseased glomeruli and decreased the number of CD8^+^ cells in the glomeruli in anti-glomerular basement membrane (GBM) crescentic glomerulonephritis. Moreover, the urinary protein level was diminished after bezafibrate treatment, in parallel with the attenuation of glomerular injury (Saga et al., 2005). Plasma free fatty acid and triglyceride levels were elevated in relation to a decrease in PPARα expression in high-fat diet (HFD) models. In these models, treatment with fenofibrate increased PPARα expression, prevented HFD-induced renal lipotoxicity, reduced oxidative stress and lipid accumulation in the glomeruli, and prevented the development of albuminuria and glomerular fibrosis (Tanaka et al., 2011; Chung et al., 2012). Taken together, these findings suggest that PPARα may be a novel therapeutic target for the treatment of kidney disease.

## PPARβ/δ

PPARβ/δ plays a key role in a number of biological processes, including fertility, lipid metabolism, bone formation, mast cell immunity, skin and brain development, and tumorigenesis (Wu et al., 2009). Although PPARβ/δ mRNA has been detected in almost all tissues and cells examined, it is relatively abundant in the kidney, with ubiquitous expression in all nephron segments, including glomerular mesangial cells, medullary interstitial cells, and stromal cells (Guan et al., 1997). However, the role of PPARβ/δ in the kidney has not been investigated in detail.

### PPARβ/δ and Acute Kidney Injury

Letavernier et al. reported that PPARβ/δ-knockdown mice exhibited much greater kidney dysfunction and exacerbated injury compared to their wild-type counterparts after I/R injury. PPARβ/δ may protect the kidney against I/R injury by activating the antiapoptotic Akt signaling pathway and increasing the spread of tubular epithelial cells (Letavernier et al., 2005). PPARβ/δ agonist treatment has also been shown to attenuate renal dysfunction, leukocyte infiltration, and the formation of interleukin (IL)-6 and tumor necrosis factor-α (TNF-α) in the diabetic kidney during I/R injury. The expression of suppressor of cytokine signaling-3, which plays important roles in the cytokine-activated signaling pathway, was increased after PPARβ/δ agonist treatment in I/R injury models (Collino et al., 2011).

### PPARβ/δ and Diabetic Nephropathy

The expression of PPARβ/δ in the kidney was downregulated in type 1 diabetic Akita and OVE26 mice, which might have been associated with decreased FAO and increased renal triglyceride accumulation (Proctor et al., 2006). The expression of PPARβ/δ was found to be increased in renal medullary interstitial cells under hypertonic conditions, with the overexpression of PPARβ/δ protecting cultured medullary interstitial cells from hypertonicity-induced cell death. These results indicate that PPARβ/δ is an important survival factor for medullary interstitial cells under hypertonic conditions in the renal medulla (Han et al., 2011).

### PPARβ/δ and Chronic Kidney Disease

PPARβ/δ attenuates kidney injury through inhibiting inflammatory and immune system. In a mouse model of protein-overload nephropathy, mice receiving the PPARβ agonist GW501516, developed less severe tubulointerstitial lesions, macrophage infiltration, and decreased mRNA expression of monocyte chemotactic protein (MCP-1) and TNFα. *In vitro*, results of the study showed that GW501516 attenuated MCP-1 expression *via* direct inhibition of the TGF-β activated kinase (TAK1)-NF-κB pathway, a common signaling pathway of inflammatory (Yang et al., 2011). PPARβ/δ has a pivotal role in maintain self-tolerance. PPARβ/δ-deficient female mice decreased expression of opsonins such as complement component-1qb (C1qb), which resulted impairment of apoptotic cell clearance and reduction in anti-inflammatory cytokine production. These mice were much more likely to develop autoimmune kidney disease, a lupus-like autoimmunity (Mukundan et al., 2009). Treatment of lupus mice with PPARβ/δ agonist reduced incidence of hypertension, endothelial disfunction, renal inflammation, and organ damage of mice, which was associated with decreased plasma anti-double-stranded DNA autoantibodies and anti-inflammatory, antioxidant effects (Romero et al., 2017).

## PPARγ

PPARγ is constitutively expressed throughout the kidney, predominantly in the distal medullary collecting ducts, and at low levels in many other nephron segments, such as the proximal tubules and renal vasculature (Yang et al., 2012; Zhou et al., 2013). In accordance with the results of other studies, PPARγ expression has also been reported in cultured glomerular mesangial cells, podocytes, and proximal epithelial cells (Zhang and Guan, 2005). Accumulating evidence has revealed the renoprotective effects of PPARγ activation (Doi et al., 2007; Miyazaki et al., 2007; Corrales et al., 2018).

### PPARγ and Acute Kidney Injury

PPARγ agonists were reported to protect the kidney against I/R injury by inhibiting I/R injury-induced diffuse tubular necrosis and acute inflammation (Reel et al., 2013), and reducing nitric oxide plasma levels, ED-1^+^ cell infiltration, and cleaved caspase-3 expression (Betz et al., 2012). A recent study showed that the PPARγ agonist pioglitazone decreased the expression of NF-κB-related proteins and the mRNA expression of inflammatory cytokines, including TNF-α and monocyte chemotactic protein-1 (MCP-1) in a renal I/R model (Zou et al., 2021). These data suggest that PPARγ agonists may be helpful in reducing renal I/R injury because of their anti-inflammatory, antioxidant, and anti-apoptosis effects. Moreover, PPARγ agonists have been found to increase AMP-activated protein kinase phosphorylation, inhibit p62 and cleaved caspase-3/8 protein expression, reduce cell apoptosis, and activate two autophagy-related proteins, LC3 II and Beclin-1, in the kidneys and proximal tubular cells of rats with an I/R injury (Chen et al., 2018; Xi et al., 2019). Therefore, PPARγ agonists exert renoprotective effects *via* the activation of autophagy. The PPARγ agonist pioglitazone protects against histological alterations in the kidney and ameliorates decreases in glutathione (GSH) and ascorbic acid levels induced by cisplatin treatment by preventing a decline in antioxidant status (Jesse et al., 2014). Pioglitazone can also decrease the expression of NF-κB p65 target genes (e.g., IL-6, IL-1β, and TNF-α) and inhibit histological injury and inflammatory cell infiltration in rats with cisplatin-induced AKI (Zhang et al., 2016). Medic et al. reported that pioglitazone reduced serum urea and creatinine levels, as well as the urinary level of kidney injury molecule-1, thus mitigating histological injury in response to gentamicin-induced kidney injury in rats (Medic et al., 2019).

Cyclooxygenase-2 (COX-2) is an inducible enzyme, which is constitutively expressed and highly regulated in response to alterations in intravascular volume (Rios et al., 2012). The protective effect of COX-2 on renal vascular function was associated with prostacyclin signaling through PPARβ/δ. PPARβ/δ activation conferred renal vasodilatory effects by regulating COX-2 and offered a potential strategy for treatment of acute renal failure (Kirkby et al., 2018). PPARα or PPARγ agonists (fenofibrate, rosiglitazone) lowered blood pressure through anti-inflammatory effect by reducing COX-2 expression in the kidney, which may be one of the indirect mechanisms of renal protection of PPARs (Bae et al., 2010; Lee et al., 2011). More researches are needed to ascertain the role of PPARs and COX-2 in renal disease.

### PPARγ and Diabetic Nephropathy

PPARγ is upregulated in the presence of high glucose in HK-2 cells. Activation of PPARγ reverses G1 phase cell cycle arrest and suppresses high-glucose-induced TGF-β and MCP-1 levels in these cells (Panchapakesan et al., 2004). Therefore, PPARγ has been postulated to be involved in the pathogenesis of DN. Stimulation of PPARγ may protect against the development of DN. PPARγ agonist treatment has been reported to be effective in improving microalbuminuria, intrarenal nitric oxide bioavailability, and protecting renal function in patients with DN (Bakris et al., 2003; Grossman, 20032003; Tang et al., 2010; Pistrosch et al., 2012). In animal experiments, PPARγ agonists were shown to improve the effects of kidney injury by preventing mesangial expansion, glomerulosclerosis, tubulointerstitial inflammation and fibrosis, and tubular dilation and atrophy, with partial improvements observed after the downregulation of renal disintegrin and metalloprotease-17 as well as angiotensin-converting enzyme-2 shedding (Ohga et al., 2007; Bilan et al., 2011; Chodavarapu et al., 2013). The PPARγ agonist rosiglitazone, which induces PGC-1α expression, may ameliorate podocyte impairment, GBM thickening, and kidney fibrosis in DN (Zhang et al., 2018). The PPAR Pro12Ala gene polymorphism was shown to be significantly associated with a decreased risk of developing DN (Tönjes and Stumvoll, 2007). It has been reported that telmisartan, a weak PPARγ agonist, can slow the progression of DN (Matsui et al., 2007). The PPARα/γ dual agonist tesaglitazar not only improved lipid metabolism and increased adiponectin levels but also prevented albuminuria and renal glomerular fibrosis in diabetic mice (Yang et al., 2009). Another study reported a similar result, that a combination of low doses of the PPARα agonist fenofibrate and the PPARγ agonist rosiglitazone attenuated diabetic kidney injury to a greater extent than did either drug alone (Arora et al., 2010).

PPARγ null mice showed increased glucosuria and albuminuria in 3 weeks old. With age the mice developed renal insufficiency, advance of type 2 diabetes, and anti-phospholipid syndrome (APS), an autoimmune disorder associated with glomerular injury and microthrombi. The results reflected PPARγ activities in systemic metabolic hemostasis, and in the immune and inflammatory system (Toffoli et al., 2017).

### PPARγ and Chronic Kidney Disease

There is a growing body of evidence showing that the activation of PPARγ plays a protective role in renal interstitial fibrosis disease. The expression of PPARγ is increased in glomeruli in a substantial proportion of patients with chronic kidney disease (CKD), particularly in macrophages, podocytes, and some parietal epithelial cells (Revelo et al., 2005; Paueksakon et al., 2142). PPARγ activation can delay the progression of CKD by inducing klotho restoration (Lin et al., 2017) and inhibiting Wnt signaling-mediated fibrogenesis (Maquigussa et al., 2018). PPARγ in renal tubular epithelial play an important role of maintaining the normal epithelial phenotype and opposing fibrogenesis (Zhao et al., 2016). In an open-label randomized crossover study in nondiabetic obese patients with proteinuric CKD, rosiglitazone treatment was shown to decrease proteinuria (Kincaid-Smith et al., 2008). In a randomized, double-blind, placebo-controlled study, rosiglitazone was also shown to lower the homeostasis model assessment score, an indicator of insulin sensitivity, in patients with CKD (Chan et al., 2011). Rosiglitazone attenuated the progression of hyperuricemic nephrophathy rat model through inhibiting TGF-β and NF-κB signaling, suppressing epithelial-to-mesenchymal transition (EMT), reducing inflammation, and lowered serum uric acid levels (Wang et al., 2020). The PPARγ agonist troglitazone ameliorated both glomerulosclerosis and aortic medial thickening in spontaneously hypertensive rats subjected to 5/6 nephrectomy (Yoshida et al., 2001). Troglitazone also attenuated renal interstitial fibrosis and inflammation in the model of unilateral ureteral obstruction (UUO) through reduction of TGF-β expression (Kawai et al., 2009). Another PPARγ agonists, pioglitazone, reduced renal fibrosis and its progression. It was recently demonstrated that pioglitazone in TGFβ transgenic mice inhibited the renal mRNA expression of all the profibrotic effectors, and TGFβ-STAT3 and TGFβ-EGR1 transcriptional activation pathways (Nemeth et al., 2019). Pioglitazone treatment of male Zucker diabetic fatty (ZDF) rats ameliorated diabetic kidney disease, improved renal blood flow and renal fibrosis, which was associated with lower renal expression of Twist-1, an evolutionarily conserved protein that can accelerate renal EMT and interstitial fibrosis (Wang et al., 2019). Moreover, similar results have been confirmed *in vitro*, with thiaziolidinediones (TZD) shown to prevent increases in TGF-β and extracellular matrix components in cultured human mesangial cells (Maeda et al., 2005) and inhibit mesangial cell and fibroblast proliferation (Nicholas et al., 2001; Zafiriou et al., 2005). Activation of PPARγ by rosiglitazone attenuated primary renal fibrolasts proliferation by suppressing AKT phosphorylation and skp2 production (Lu et al., 2016). PPARγ agonists were reported to delay the progression of polycystic kidney disease in a rat model by inhibiting cell proliferation and fibrosis (Yoshihara et al., 2011). PPARγ agonists were also found to inhibit renal interstitial macrophage infiltration, downregulate the expression of downstream target genes, and upregulate bone morphogenetic protein-7 expression, eventually blocking renal fibrosis (Lin et al., 2005).

Podocyte damage is the crucial step in the pathogenesis of CKD and progression of end stage renal disease (ESRD). Some studies have focused on the role of PPARγ activation in preventing podocyte injury. It was reported that PPARγ agonist protected podocytes in acute nephric syndrome, which was dependent partially on restoration of podocyte structure (Zuo et al., 2012). Rosiglitazone completely restored the reduced nephrin expression, and prevented MtD and oxidative stress in podocytes exposed to the mineralocorticoid aldosterone (Aldo). It was suggested that rosiglitazone might protect podocytes from injury by improving mitochondrial function (Zhu et al., 2020-31). Pioglitazone decreased puromycin aminonucleoside (PAN)-induced podocyte apoptosis and necrosis, while restoring podocyte differentiation (Kanjanabuch et al., 2007). PPARγ activation in the podocyte seems to be a key protective response after injury. PPARγ agonists might be effective in the treatment of CKD by protecting podocytes.

PPARγ activation plays a potently role in preservation of renal function of kidney allografts. Rosiglitazone has the immunosuppressive, antifibrotic, antiproliferative, anti-inflammatory actions, which are the leading causes of chronic allograft failure. It was reported that rosiglitazone treatment reduced serum creatinine, albuminuria, chronic allograft damage in the rat renal transplantation models. Meanwhile, the deposition of extracellular matrix proteins such as collagen, fibronectin, decorin was lowered (Kiss et al., 2010). Administration of rosiglitazone also reduced proteinuria and decreased interstitial collagen deposition in renal allograft transplantation model. It was suggested that rosiglitazone attenuated the development of chronic renal allograft dysfunction *via* inhibition of TGF-β and NF-κB pathway activation, the renal EMT, and inflammation (Deng et al., 2019).

Klotho is an anti-aging protein mainly expression in the kidney. A decreased expression of Klotho has been reported in aging and CKD. Therapeutic approaches to stimulate Klotho expression in CKD can exert vasculo-protective effects (Buchanan et al., 2020). The Klotho gene has two upstream non-canonical PPARγ binding sites. Influencing the PPARγ pathway might result in an increased renal tubular Klotho mRNA and protein expression (Zhang et al., 2008). PPARγ activation was attributed to increased renal Klotho expression and reduced oxidative stress, which effectively ameliorated the age-related nephrosclerosis in ApoE-null mice (Shen et al., 2018). Acetylation of PPARγ could prevent Klotho loss, and attenuate renal damage in CKD mouse model consequentially (Lin et al., 2017).

## Conclusion

The kidney is a highly metabolic organ and consumes a large amount of energy to maintain fluid and electrolyte homeostasis. All three PPAR isotypes perform complementary physiological functions and may confer therapeutic benefits in kidney disease. The activation of PPAR isotypescan result in distinct biological processes in kidney disease. Agonists of PPARs show considerable promise for the treatment of AKI, DN, glomerulonephritis, and CKD. However, some undesirable severe side effects of PPAR agonists have been reported. For example, they can result in increased serum levels of creatinine and cystatin C, and potentially decrease the estimated glomerular filtration rate and creatinine clearance (Hiukka et al., 2010). Therefore, these agonists should be used with caution in clinical therapy for kidney diseases. Further large-scale, prospective, randomized trials are necessary to evaluate the effects of these agonists on renal outcomes in patients with kidney disease.
